# Distinct expression pattern of the full set of secreted phospholipases A_2_ in human colorectal adenocarcinomas: sPLA_2_-III as a biomarker candidate

**DOI:** 10.1038/sj.bjc.6604184

**Published:** 2008-01-22

**Authors:** C M Mounier, D Wendum, E Greenspan, J-F Fléjou, D W Rosenberg, G Lambeau

**Affiliations:** 1 Institut de Pharmacologie Moléculaire et Cellulaire, CNRS-UNSA UMR6097, Sophia Antipolis, Valbonne, France; 2 INSERM-UMR S538, Université Pierre et Marie Curie – Paris6, Paris, France; 3 AP-HP, Hôpital Saint-Antoine, Service d'Anatomie Pathologique, Paris, France; 4 Center for Molecular Medicine, University of Connecticut Health Center, Farmington, CT, USA

**Keywords:** colon cancer, secreted phospholipase A_2_, gene expression, qPCR, immunohistochemistry, biomarker

## Abstract

Recent studies suggest that secreted phospholipases A_2_ (sPLA_2_s) represent attractive potential tumour biomarkers and therapeutic targets for various cancers. As a first step to address this issue in human colorectal cancer, we examined the expression of the full set of sPLA_2_s in sporadic adenocarcinomas and normal matched mucosa from 21 patients by quantitative PCR and immunohistochemistry. In normal colon, *PLA2G2A* and *PLA2G12A* were expressed at high levels, *PLA2G2D*, *PLA2G5*, *PLA2G10* and *PLA2G12B* at moderate levels, and *PLA2G1B*, *PLA2G2F* and *PLA2G3* at low levels. In adenocarcinomas from left and right colon, the expression of *PLA2G3* was increased by up to 40-fold, while that of *PLA2G2D* and *PLA2G5* was decreased by up to 23- and 14-fold. The variations of expression for sPLA_2_-IID, sPLA_2_-III and sPLA_2_-V were confirmed at the protein level. The expression pattern of these sPLA_2_s appeared to be linked respectively to the overexpression of *interleukin-8*, *defensin α6*, *survivin* and *matrilysin*, and downregulation of *SFRP-1* and *RLPA-1*, all these genes being associated to colon cancer. This original sPLA_2_ profile observed in adenocarcinomas highlights the potential role of certain sPLA_2_s in colon cancer and suggests that sPLA_2_-III might be a good candidate as a novel biomarker for both left and right colon cancers.

Sporadic colorectal cancer is a major human health concern in industrialised countries and is the third leading cause of mortality by cancer. The accumulation of genetic alterations in several key genes (*APC*, *K-ras*, *DCC* and *p53*) has been associated with tumour development (Fearon and Vogelstein, 1990). In particular, the activation of the Wnt/*β*-catenin/Tcf-4 pathway is known to play a key role in colon tumorigenesis, leading to overexpression of a large number of target genes, including *c-myc*, *cyclin D1*, *MMP-7*, *ITF-2*, *Il-8*, *gastrin*, *uPAR*, *cryptdin/defensin* and *EPR-1* genes (Kolligs *et al*, 2002; Levy *et al*, 2002). Moreover, the participation of tumour modifier genes may also contribute to pathogenesis, providing a basis to individual genetic predispositions, in addition to environmental and diet influences (Wiesner *et al*, 2003; Yan *et al*, 2004; Rakoff-Nahoum and Medzhitov, 2007). A comprehensive characterisation of such tumour modifier genes must take into account the stage of cancer and the location of the tumour within the large intestine, that is, ‘right’ or ‘left’ colon. This information may lead to a better understanding of the molecular events involved in colorectal cancer, and may provide new therapeutic targets to improve patient treatment outcomes (Williams *et al*, 2003; Barrier *et al*, 2005). It is also of interest to investigate the expression of such genes to identify new biomarkers of human colorectal cancers for early diagnosis and prognosis (Williams *et al*, 2003; Barrier *et al*, 2005).

In humans, 10 secreted phospholipases A_2_ (sPLA_2_s) have been described in the last decade, and the analysis of their functions in physiological and pathophysiological conditions is under intense investigation. Human sPLA_2_s have been classified into groups IB, IIA, IID, IIE, IIF, III, V, X, XIIA and XIIB according to their structural properties (Laye and Gill, 2003; Rouault *et al*, 2003; Murakami *et al*, 2005; Cummings, 2007). Several of these proteins have recently been proposed either as biomarkers of pathologies (Smith *et al*, 2003; Mallat *et al*, 2005; Wootton *et al*, 2007) or as therapeutic targets (Laye and Gill, 2003; Cummings, 2007; Henderson *et al*, 2007). These enzymes have the capacity to generate biologically active lipid mediators such as lysophosphatidic acid (LPA) and arachidonic acid (AA), which can be further converted into prostaglandin E_2_ (PGE_2_). These lipid mediators are known to be involved in cell proliferation, cell migration, angiogenesis, and are likely to play a role in the initiation and/or progression of colorectal cancer (Morioka *et al*, 2000; Krause and DuBois, 2001; Laye and Gill, 2003; Mills and Moolenaar, 2003; Murakami *et al*, 2005). AA can also participate in apoptosis by activating sphingomyelinases and ceramide production (Dong *et al*, 2005; Ilsley *et al*, 2005).

Secreted phospholipases A_2_ have been recently proposed as targets for anticancer drugs (Laye and Gill, 2003; Cummings, 2007), and there is increasing evidence for their involvement in various human cancers. Indeed, the expression levels of sPLA_2_-V and sPLA_2_-X are modified in human lung cancer (Masuda *et al*, 2005b), and those of sPLA_2_-V and sPLA_2_-XIIA in human ovarian cancer (Gorovetz *et al*, 2006). In particular, the overexpression of human sPLA_2_-IIA in gastric adenocarcinoma was proposed to be related to prolonged survival and less frequent metastasis (Leung *et al*, 2002; Aggarwal *et al*, 2006). By contrast, sPLA_2_-IIA overexpression has been associated to oncogenic effects in prostate cancer (Sved *et al*, 2004) and is related to poor prognosis (Graff *et al*, 2001). The polymorphism of the sPLA_2_-IIA gene, *PLA2G2A*, is also associated to some phenotypic features of patients with familial adenomatous polyposis (Yanaru-Fujisawa *et al*, 2007). More recently, human sPLA_2_-XIIB was shown to be upregulated in 50% of patients with hepatitis C virus-associated hepatocellular carcinoma (Smith *et al*, 2003). Finally, a protective role for sPLA_2_-IIA against *Apc*^*Min*^-induced intestinal tumours has been established in mice (MacPhee *et al*, 1995; Cormier *et al*, 1997). However, data on sPLA_2_ and human cancers are still sparse, and often rely on only one or a few sPLA_2_s.

In this study, we have analysed the expression pattern of the full set of sPLA_2_s in tumour *vs* normal matched mucosa from patients with adenocarcinomas located in the left and right colon. Variation in the expression of a number of other genes associated with colon tumorigenesis and/or inflammation was also examined to establish possible gene coregulations. Our data provide the first comprehensive analysis of the expression pattern of all sPLA_2_s in normal human colon mucosa as well as adenocarcinomas. These data further support the fact that several sPLA_2_s may contribute to the initiation, progression or modulation of colon tumorigenesis, and may provide new potential tumour markers for this disease.

## MATERIALS AND METHODS

### Human tissues and RNA isolation

Fourteen colon adenocarcinoma specimens from the left colon (descending part of the colon) and seven colon adenocarcinoma specimens from the right colon (ascending part of the colon) were obtained from surgical resections according to the French and American institutional guidelines. No specimen was from rectum. The patients did not receive chemotherapy or radiotherapy prior to surgery. The specimens were from 42- to 85-year-old patients (median age: 71 years), 14 men and 7 women. Samples were well (3 out of 21), moderately (14 out of 21) or poorly (4 out of 21) differentiated sporadic adenocarcinomas, with pTNM classification ranging pT2–4 N0–2 M0–1. Neither adenoma nor Duke'D adenocarcinoma was examined in this study. All samples had an expression of hMLH1 and hMSH2 unchanged as evaluated by immunohistochemistry and quantitative PCR (qPCR) (data not shown). Tissues from the non-necrotic part of the tumour and from distant normal mucosa were snap frozen in liquid nitrogen and stored at −80°C. Each tissue sample (20–100 mg) was mixed with 700 *μ*l of lysis buffer plus *β*-mercaptoethanol (nucleospin RNA II kit; Macherey-Nagel, Hoerdt, France) in a green-cap tube containing Lysing Matrix D (Q-BIOgene, Illkrich, France), and tissue disruption was achieved with the fast prep instrument (FP220A; Q-BIOgene). Total RNA was then isolated using the nucleospin RNA II kit, including DNase treatment. RNA concentration was determined by OD_260_ and RNA quality was evaluated by analysis on an Agilent Bioanalyzer (Agilent Technologies, Les Ulis, France).

### Quantitative PCR

**Figure 1:**
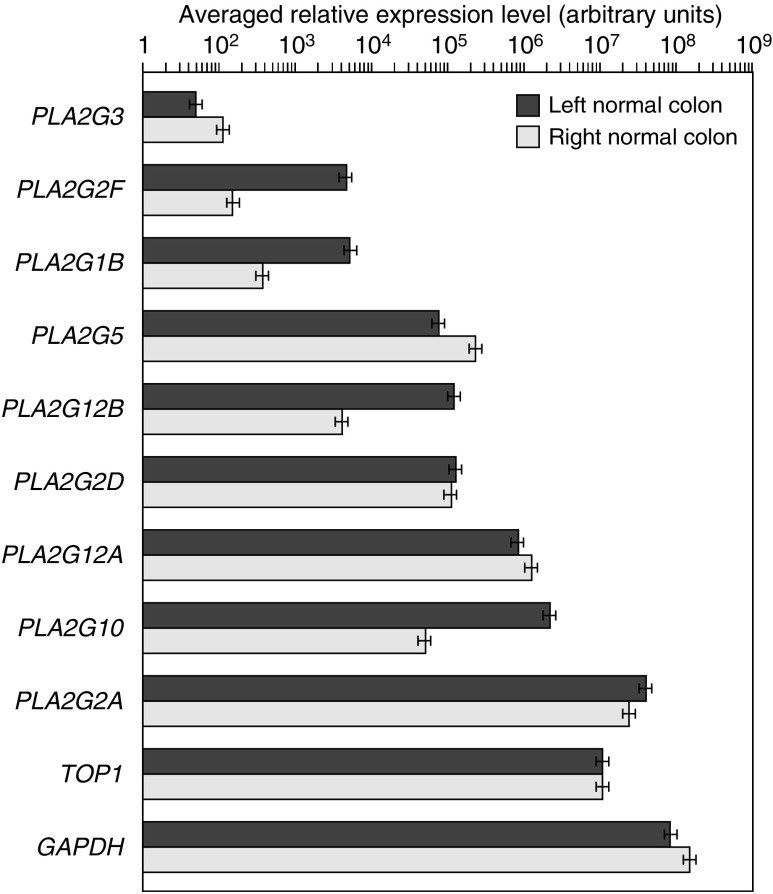
Expression level of sPLA_2_ genes in human colon normal mucosa. The relative averaged expression level of all sPLA_2_ genes and the two reference genes used in this study (*TOP1* and *GAPDH*) is shown for the 14 samples from left colon and the 7 samples from right colon. Arbitrary units are used (see Materials and Methods).

**Figure 2:**
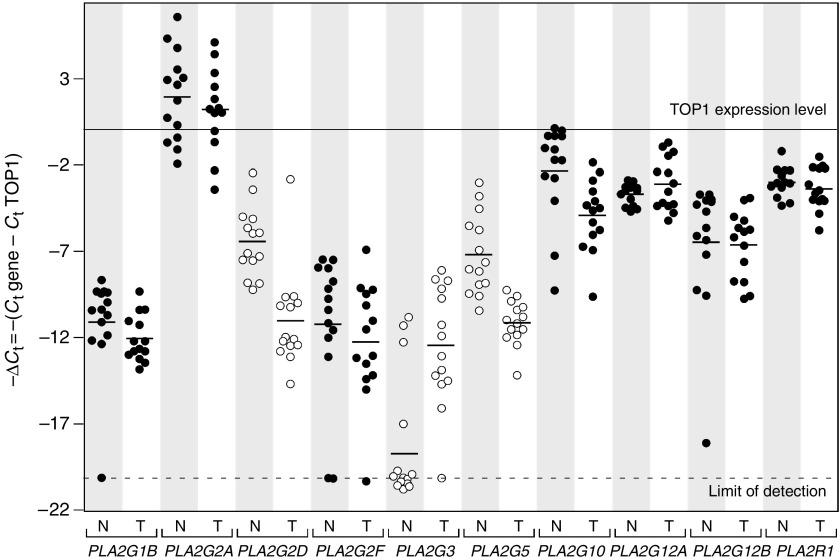
Expression level of sPLA_2_ genes in human adenocarcinomas and normal matched mucosa. The expression level of sPLA_2_ genes and of the M-type sPLA_2_ receptor (*PLA2R1*) was measured in adenocarcinomas and normal matched mucosa for the 14 left-sided samples. The data were obtained after normalisation with *TOP1* used as reference gene and using the formula −Δ*C*_t_=*C*_t_ gene of interest−*C*_t_ TOP1. N, normal tissues; T, tumour tissues.

**Figure 3:**
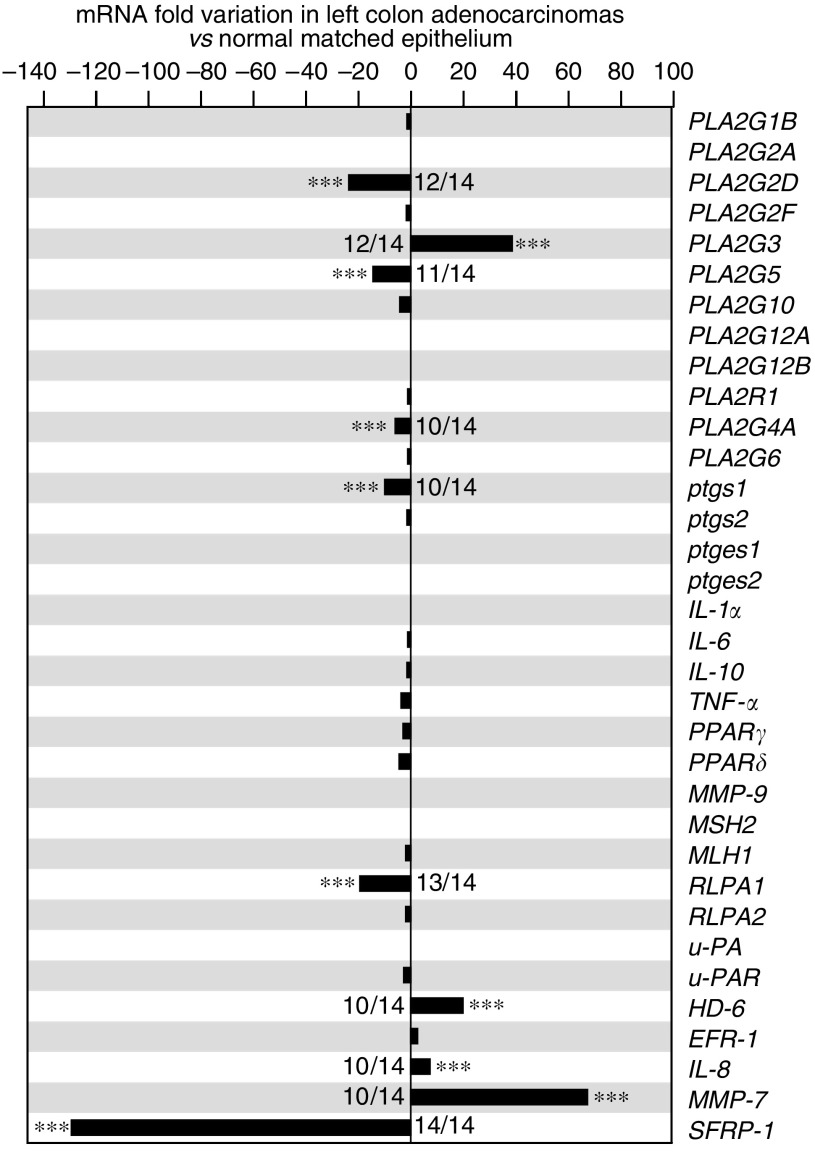
Expression level of sPLA_2_, tumour-related and inflammation-related genes in human left colon adenocarcinomas. After normalisation with the *TOP1* reference gene, the expression level for each gene in the tumour was compared to that in the normal matched mucosa. The zero value indicates no variation, a positive value indicates an increased expression level in tumour *vs* normal tissue and a negative value indicates a decreased expression level in tumour *vs* normal tissue (see Materials and Methods). The incidence of variation in the different samples is indicated.

**Figure 4:**
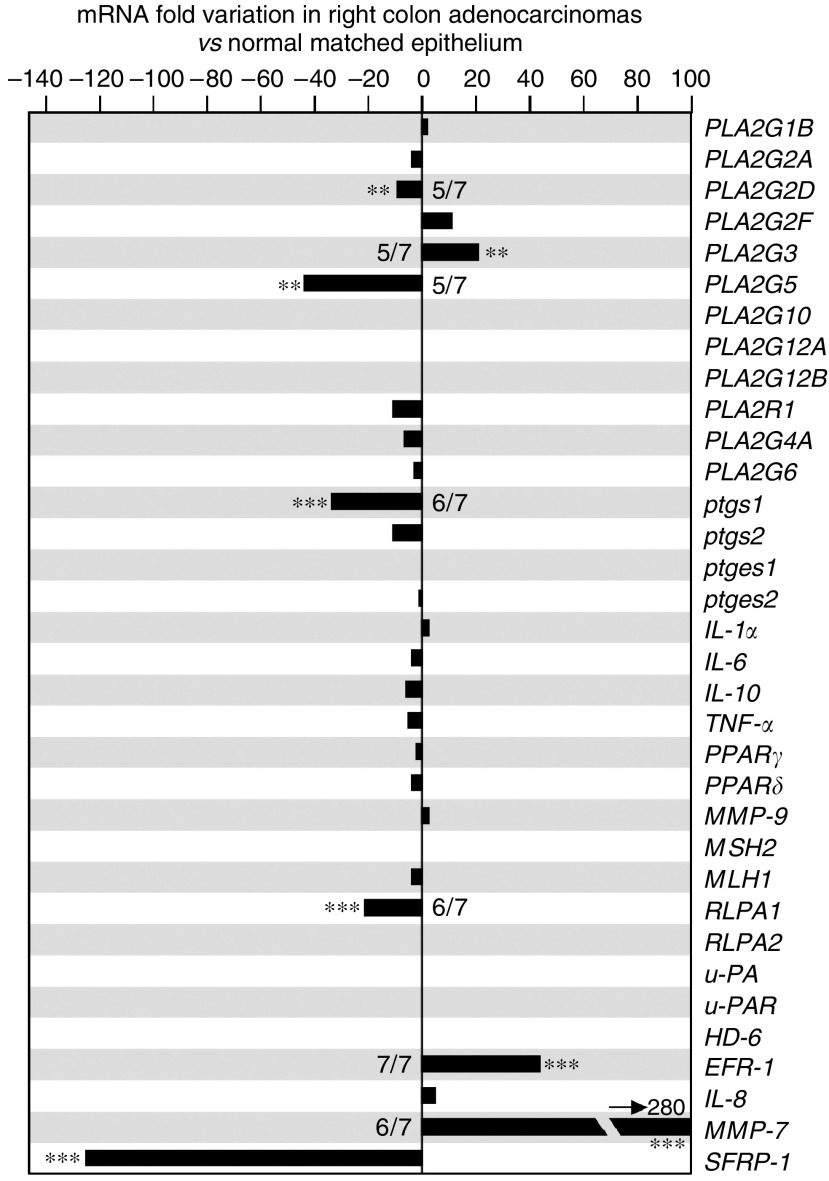
Expression level of sPLA_2_, tumour-related and inflammation-related genes in human right colon adenocarcinomas. Same legend as Figure 3.

**Figure 5:**
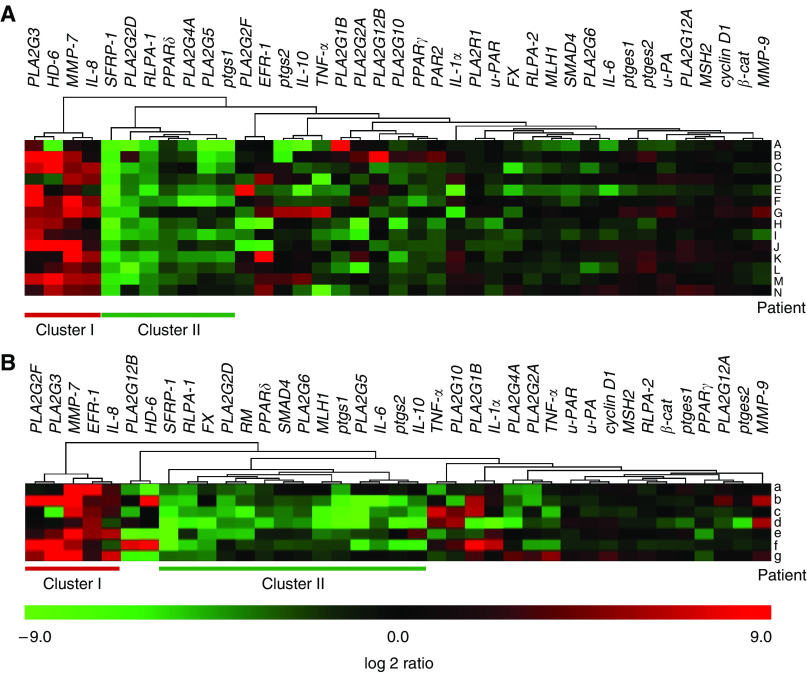
Hierarchical clustering of genes. Heatmaps comparing the log 2 ratio between the expression level in adenocarcinomas and normal epithelium for the 14 patients with left-sided adenocarcinomas (**A**) and for the 7 patients with right-sided adenocarcinomas (**B**) are shown. The distance corresponds to an Euclidean distance calculated using the Mev3.1 software.

First-strand cDNA was synthesised from 5 *μ*g of total RNA using 100 U of MMLV reverse transcriptase (#M170A; Promega, Charbonnières-les bains, France) in a final volume of 50 *μ*l with 500 ng of random primers (#C118A; Promega). Quantitative PCR was carried out in 96-well ABgene plates using the GENEAMP 5700 sequence Detection System apparatus (Applied Biosystems, Courtaboeuf, France) with the qPCR Master Mix Plus for SYBR® Green I (Eurogentec, Angers, France). All reactions were performed in a total volume of 16 *μ*l and contained 50 ng of reverse transcribed RNA (based on the initial RNA concentration) and 250 nM of each primer set. The primer sets were designed using the Primer Express program from Applied Biosystems for the following human genes: *PLA2G2A* (NM_000300), sPLA_2_-IID gene (*PLA2G2D*) (NM_012400), sPLA_2_-IIE gene (*PLA2G2E*) (NM_0145891), sPLA_2_-IIF gene (*PLA2G2F*) (NM_022819), sPLA_2_-III gene (*PLA2G3*) (NM_015715), sPLA_2_-V gene (*PLA2G5*) (NM_000929), sPLA_2_-X gene (*PLA2G10*) (NM_003561), sPLA_2_-XIIA gene (*PLA2G12A*) (BC_017218), sPLA_2_-XIIB gene (*PLA2G12B*) (NM_032562), iPLA_2_-VIB gene (*PLA2G6*) (NM_003560), *ptgs1* (BC_029840), COX-2 gene (*ptgs2*) (NM_000963), *ptges1* (NM_004878), *ptges2* (NM_198797), *Il-1α* (NM_000575), *Il-6* (NM_000600), *Il-10* (NM_57627), *PPARγ* (NM_138712), *PPARδ* (NM_006238), *u-PA* (NM_002658), *u-PAR* (NM_002659), *IL-8* (NM_000584), *EPR-1* (NM_002219), *MMP-7* (NM_002423), *SFRP-1* (NM_003012), *PLA2R1* (NM_007366), *TNF-α* (NM_000594), *MMP-9* (NM_004994), *MSH2* (NM_000251), *MLH1* (NM_000249). Most primer sets were designed to span an intron in order to avoid amplification from potential traces of genomic DNA in the total RNA preparations. Only the primer sets for *MLH1*, *uPAR*, *EPR-1*, *MMP-7* and *bcl-2* genes were not spanning an intron. For primer sets spanning an intron, we checked that no amplification signal was obtained using human genomic DNA as template in the qPCR (data not shown). The sequences of the designed primer sets are available on request. We used Qiagen commercial primer sets for cPLA_2_-IVA gene (*PLA2G4A*) (ref. QT00085813) and sPLA_2_-IB gene (*PLA2G1B*) (ref. QT00000637). We used published primer sets for *RLPA-1* and *RLPA-2* genes (Shida *et al*, 2004) and *HD-6* gene (Andreu *et al*, 2005). The efficiency and specificity of each primer sets were validated using either serial dilutions of cloned human sPLA_2_ cDNAs or mixed human tissue cDNA for the other genes. Moreover, when enough total RNA was collected, negative controls without added reverse transcriptase were performed. Thermal cycling was performed at 95°C for 10 min, followed by 40 cycles comprising each a denaturation step at 95°C for 15 s, and an annealing/extension step at 60°C for 1 min. Amplification of the appropriate product was verified by analysing the dissociation curve that was obtained after PCR with the following steps: 15 s at 95°C, 20 s at 60°C, and then a slow ramp of 20 min from 60 to 95°C. The abundance of the mRNA target was calculated relative to the expression of the reference gene *TOP1* and is expressed as 2^Δ*C*_t_^, where Δ*C*_t_=*C*_t_ (gene of interest)−*C*_t_ (TOP1). The choice of *TOP1* as a reference gene was determined using a Human GeNorm kit (PrimerDesign Ltd, Southampton, UK), allowing the determination of the best reference gene among 12 widely used reference genes. The data were also validated using *GAPDH* as a reference gene (data not shown). When the relative level of expression was plotted for normal mucosa (n) (Figure 1), we used the formula 2^20-Δ*C*_t_^, with Δ*C*_t_=*C*_t_(n)gene−*C*_t_(n)TOP1. The *C*_t_(n) values for TOP1 were typically around 20. When the expression of each gene in normal mucosa *vs* tumours (Figure 2 , 3 and 4) was plotted, the comparative *C*_t_ method (detailed in the ABI Prism 7700 Sequence Detection System User Bulletin no. 2) was used to determine the relative quantities of mRNA, and we changed the sign in order to get the lowest level of expression at the bottom and the highest level of expression at the top of the *y* axis, that is, −Δ*C*_t_=−(*C*_t_ gene of interest−*C*_t_ TOP1). When the expression of each gene was compared between the tumour (t) and the matched normal mucosa (n), the decrease or increase factor *ϕ* in the tumour *vs* the normal mucosa (Figure 5A and B) was calculated with the formula ϕ= 2^*I*Δ*C*_t_(n)-Δ*C*_t_(t)*I*^-1, with Δ*C*_t_(n)=*C*_t_(n)gene−*C*_t_(n)TOP1, Δ*C*_t_(t)=*C*_t_(t)gene−*C*_t_(t)TOP1, and *I* the absolute value of the term Δ*C*_t_(n)−Δ*C*_t_(t).

### Immunohistochemistry

All experiments were performed using paraffin-embedded tissues from the same patients as those used for the qPCR experiments. The expression of sPLA_2_-IID, sPLA_2_-III, sPLA_2_-V and COX-2 proteins was analysed in tumours and normal matched tissues from three to four patients. Consecutive 4-*μ*m tissue sections were deparaffinised in xylene and rehydrated in graded alcohol dilutions. Immunolabelling was performed using avidin–biotin–peroxidase technique (Vectastain ABC kit; Vector, Burlingame, CA, USA). Before immunostaining, endogenous peroxidase activity was inhibited with 0.1% hydrogen peroxide in methanol for 30 min. Colour development was achieved with 3-amino-9-ethyl-carbazole, and sections were finally counterstained with haematoxylin. Specific rabbit polyclonal antibodies against recombinant human sPLA_2_-IID, sPLA_2_-III and sPLA_2_-V were produced as described (Haas *et al*, 2005), and used at working dilutions of 1/300, 1/250 and 1/100, respectively. All anti-sPLA_2_ antibodies were tested for specificity towards the various sPLA_2_s and were shown to be highly specific for each human sPLA_2_ (Haas *et al*, 2005). Negative controls were performed by omission of the primary antibody. To further check for the specificity of labelling with sPLA_2_-IID-, sPLA_2_-III- and sPLA_2_-V-specific antibodies, competition experiments were performed in which the antibody was preincubated with the corresponding purified recombinant sPLA_2_ used for immunisation, prior to covering the tissue slides. The competition was performed by preincubating the relevant antibody solution with 200 nM of human recombinant sPLA_2_-III or 100 nM of human recombinant sPLA_2_-IID and sPLA_2_-V for 1 h at room temperature. Purified recombinant proteins were obtained as described (Rouault *et al*, 2007). Human COX-2 antibodies were from Cayman (Montigny-le Bretonneux, France) (ref. 160112, working dilution 1/500), human MLH1 antibodies were from BD Pharmingen (Le Pont-de-Claix, France) (clone G168-178, working dilution 1/100), and human MSH2 antibodies were from Calbiochem (Darmstadt, Germany) (clone FE-11, working dilution 1/125).

### Statistics

We determined that the sample data from left and right colons followed a Gaussian distribution using the D'agostino–Pearson normality test. Normal mucosa and tumour paired data for each gene were analysed using the Bonferroni test, and statistical significances were represented by ^*^P<0.05, ^**^P<0.01 and ^***^P<0.001. We looked for correlation between all genes examined in this study by using the Mev3.1 software and hierarchical clustering to analyse different linkages to cluster genes and samples. Euclidean distance was used to calculate the distance between two genes.

## RESULTS

### Expression levels of sPLA_2_ genes in normal human colon mucosa

We first looked at the mRNA levels for the different sPLA_2_ genes in normal mucosa and compared their relative expression levels. Our data show dramatic differences in the relative expression of sPLA_2_. The lowest gene expression level was found for *PLA2G3*, and the highest gene expression level was found for *PLA2G2A* (Figure 1). The level of *PLA2G2A* expression was particularly high. It is above that of *TOP1* and almost reaches that of *GAPDH*, which are both used as reference genes and are known to be highly expressed in many tissues, including colon. By contrast, the expression of *PLA2G3* was generally very low, in fact below the limit of detection in many samples (Figure 1). The expression levels of *PLA2G2F* and *PLA2G1B* were also low, while that of other sPLA_2_ genes were moderate. The expression of *PLA2G2E* was not detectable (data not shown). We also compared the expression levels in normal mucosa of left and right colon. A significant difference in gene expression levels was seen for *PLA2G2F*, *PLA2G1B*, *PLA2G12B* and *PLA2G10* (respectively 30-, 13-, 24- and 40-fold lower expression in right-sided normal mucosa than in left-sided normal mucosa). No or minor differences were observed in gene expression for *PLA2G2A*, *PLA2G2D*, *PLA2G3*, *PLA2G5* and *PLA2G12A* between right- and left-sided normal mucosa (Figure 1).

### Expression levels of sPLA_2_ genes in left and right colon adenocarcinomas *vs* normal human colon mucosa

We next compared sPLA_2_ gene expression in adenocarcinomas *vs* normal mucosa. The raw data for samples from the left colon are shown in Figure 2. The fold-increase or fold-decrease in the expression of each gene in adenocarcinomas *vs* normal mucosa is represented in Figure 3. Interestingly, we observed a 40-fold increase in *PLA2G3* expression in the adenocarcinomas. In contrast, we found 23- and 14-fold decreases for *PLA2G2D* and *PLA2G5*, respectively. Similar data were obtained for samples from the right colon, with a 22-fold increased expression in tumour *vs* normal mucosa for *PLA2G3*, and 10- and 44-fold reduced expression for *PLA2G2D* and *PLA2G5*, respectively (Figure 4). No variation in expression between the tumours and normal mucosa was observed for *PLA2G1B*, *PLA2G2A*, *PLA2G2F*, *PLA2G10*, *PLA2G12A* and *PLA2G12B* and for the M-type sPLA_2_ receptor (*PLA2R1*) (Figure 3 and 4). The expression of *PLA2G2E* was not detectable in any of the samples analysed (data not shown).

### Expression levels of genes involved in inflammation and tumorigenesis in left and right colon adenocarcinomas *vs* normal human colon mucosa

Since sPLA_2_s may exert a coordinate action with other genes in tumorigenesis and associated inflammation, we also examined the expression of a panel of genes known to be involved in inflammation and colorectal tumorigenesis (Figure 3 and 4). In left colon samples, we did not observe any differences in expression levels between adenocarcinomas and normal mucosa for most of the inflammation-related genes examined in this study, that is, *ptgs2*, *ptges1*, *ptges2*, *TNF-α*, *Il-1α*, *Il-6*, *Il-10*, *PPARγ*, *PPARδ*, *MMP-9* and *PLA2G6*. Only a slight decrease in the expression of *ptgs1* and *PLA2G4A* was observed. With the exception of *ptges2* and *Il-1α*, we found that the inflammation-related genes were already expressed at high levels in the normal mucosa.

We also looked at the expression of urokinase plasminogen activator (*u-PA*) and its receptor (*u-PAR*) that play a role in cell adhesion and cell migration, and are involved in late stages of tumour development, contributing to tumour cell invasion and metastatic spread (Terada *et al*, 2005). No changes were observed in *u-PA* and *u-PAR* gene expression levels.

*RLPA-1* and *RLPA-2* genes code for two distinct LPA receptors (Mills and Moolenaar, 2003). While the expression of *RLPA-2* did not change, that of *RLPA-1* was decreased by 20-fold in tumour *vs* normal matched mucosa.

*IL-8*, *EPR-1*, *HD-6* and *MMP-7* are target genes of the Wnt/*β*-cat/tcf-4 pathway (Kolligs *et al*, 2002; Levy *et al*, 2002; Wheatley and McNeish, 2005). Interestingly, a marked increase in expression was found for *Il-8*, *HD-6* and *MMP-7* expression in the tumours (8-, 22- and 70-fold, respectively). The level of *EPR-1* was not changed. *SFRP-1* codes for an antagonist of the Wnt pathway, which binds to the frizzled receptor and blocks frizzled–Wnt interaction (Suzuki *et al*, 2004). Our results show a dramatic 130-fold decrease in the expression of *SFRP-1* in the tumour.

Similar data were obtained for most genes in the right-sided samples (Figure 4). Indeed, no variation in expression was observed for most inflammatory-related genes, while a dramatic increase (280-fold) and a strong decrease (125-fold) were found for *MMP-7* and *SFRP-1*, respectively. However, in contrast to left-sided samples, we observed no variation in *HD-6* and *Il-8* expression levels and a strong increase (45-fold) in *EPR-1* expression.

### Incidence and coregulation of studied genes

We first looked at the incidence of the above variations, that is, the number of samples showing an increase or decrease in the expression level of a particular gene within the 21 patients used in this study (Figure 3, 4 and 5). The increase in *PLA2G3* expression showed a high incidence (12 out of 14 for left-sided samples and 5 out of 7 for right-sided samples), which was similar to that of *MMP-7* gene overexpression (10 out of 14 for left-sided samples and 6 out of 7 for right-sided samples). The incidence for the overexpression of *HD-6* and *Il-8* was lower, since these two genes were not significantly upregulated in right-sided samples. The decrease in *PLA2G2D* and *PLA2G5* expression also showed a high incidence (12 out of 14 and 5 out of 7; and 11 out of 14 and 5 out of 7, respectively), which was similar to that of *RLPA-1* and *SFRP-1* gene expression (13 out of 14 and 6 out of 7; and 14 out of 14 and 7 out of 7, respectively). We did not observe any difference between female and male patients used in our study for the variations of expression of *PLA2G3*, *PLA2G2D* and *PLA2G5* (data not shown).

Euclidean distance is commonly used to evaluate linkages in the expression of different genes. We used this method to point out linkages between the variations in expression level of the most relevant genes. In left-sided adenocarcinomas (Figure 5A), a first cluster (cluster I) of upregulated genes was observed containing *PLA2G3* and several genes related to colon cancer such as *HD-6*, *MMP-7* and *Il-8*. A second cluster (cluster II) of several downregulated genes, including *SFRP-1*, *PLA2G2D*, *RLPA-1*, *PLA2G5*, *PLA2G4A* and *ptgs1*, was observed. These two clusters appeared most likely to discriminate adenocarcinomas from normal tissues. Remarkably, there are 3 sPLA_2_ genes out of 11 genes in these two clusters. The other genes did not show any linkage with colon tumorigenesis, since their expression levels were up- or downregulated or not changed among the different patients. When looking at right-sided samples (Figure 5B), similar patterns were observed even though the number of patients was lower.

### Protein expression levels of sPLA_2_-IID, sPLA_2_-III and sPLA_2_-V in colon adenocarcinomas and normal matched mucosa

**Figure 6:**
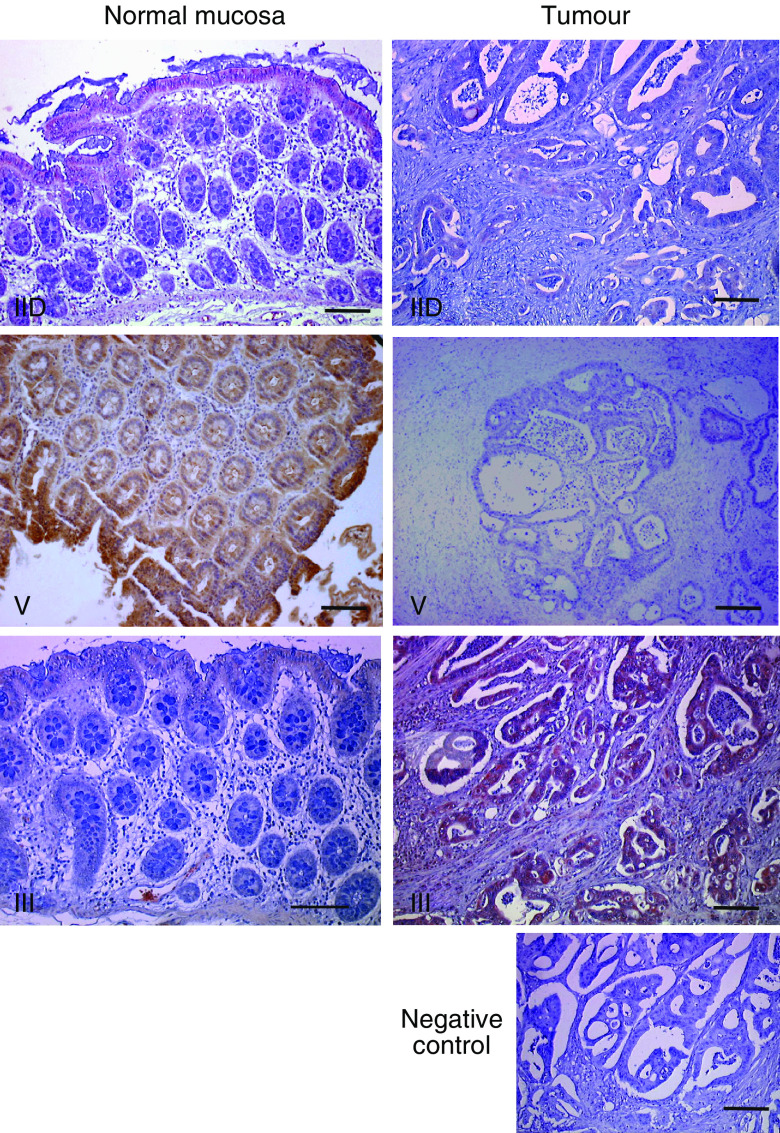
Secreted phospholipase A_2_ immunolabelling in representative colon adenocarcinomas and normal matched mucosa. For sPLA_2_-IID and sPLA_2_-V expression, a strong staining in the surface epithelium of the normal mucosa was observed, contrasting with the weak or absent staining observed in tumour cells. For sPLA_2_-III expression, a very faint staining was observed in normal epithelial cells, contrasting with the moderate to strong staining observed in tumour cells. For each sPLA_2_, a representative immunolabelling from 1 patient out of 3–4 patients analysed is shown. As shown in the bottom picture, no staining is observed when the primary antibody is omitted. Scale bar is 100 *μ*m.

To confirm that the variation of expression for *PLA2G2D*, *PLA2G3* and *PLA2G5* is also observed at the protein level, immunohistochemical analyses were performed on tissue sections from the same patients as those used for qPCR. For each sPLA_2_ protein analysed, tissue sections from three or four patients showing a significant increase or decrease of sPLA_2_ expression were selected and immunostained (Figure 6). The labelling for sPLA_2_-IID and sPLA_2_-V was markedly decreased in tumour cells compared to the normal matched epithelium, in accordance with the qPCR data. By contrast, the labelling for sPLA_2_-III was absent or very low in the normal epithelium, with a significant increase in tumour tissue. The absence of labelling in competition experiments (data not shown) or when the primary antibody was omitted (Figure 6) demonstrated that the signals observed for the three sPLA_2_ proteins were specific. In addition, immunohistochemical analysis of COX-2 showed a more intense protein expression in tumours than in normal mucosa in a subset of patients (data not shown).

## DISCUSSION

Several lines of evidence have been accumulated during the past decade to support the role of sPLA_2_s in cancer pathogenesis. Their role in human cancer, however, has not been clarified. As a first step towards addressing this issue, we have analysed the expression patterns of the full set of human sPLA_2_s in colorectal cancer tissue samples and normal matched mucosa. Our results indicate that (i) several sPLA_2_s, including sPLA_2_-IIA, sPLA_2_-X and sPLA_2_-XIIA, are highly expressed in both normal and tumour colon tissues; (ii) the expression levels of sPLA_2_-III, sPLA_2_-IID and sPLA_2_-V are dramatically altered in adenocarcinomas at both mRNA and protein levels; and (iii) their mRNA profiles are part of gene expression clusters with other genes associated with inflammation and cancer. Together, our data are further suggestive of a role of these sPLA_2_s in colorectal cancer and open the possibility that sPLA_2_s, in particular sPLA_2_-III, may provide novel cancer biomarkers.

### sPLA_2_ gene expression levels in colon adenocarcinomas and normal human colon

Colon adenocarcinomas analysed in this study are likely cancers without high microsatellite instability. Indeed, we used immunohistochemical analysis as a reliable method for screening DNA mismatch repair defects (Lindor *et al*, 2002), and found that the expression of hMLH1 and hMSH2 was unchanged. Furthermore, we analysed the normal mucosa and the tumour tissues from the right and left colon separately because of their distinct gene expression patterns (Glebov *et al*, 2003; Birkenkamp-Demtroder *et al*, 2005). We observed similar levels of expression for most sPLA_2_s in left and right normal mucosa, with *PLA2G2A* having a high expression level, *PLA2G3* being not detectable in most samples and the other sPLA_2_ genes showing intermediate levels of expression (Figure 1). Interestingly, we observed marked alterations in the expression levels of *PLA2G2D*, *PLA2G5* and *PLA2G3* in colon adenocarcinomas. In contrast, there was no difference in the expression levels of the other sPLA_2_ genes (Figure 2, 3 and 4). The absence of tumour-related alterations in the gene expression of *PLA2G2A* and *PLA2G10* in human sporadic colon cancer tissues is consistent with earlier studies (Dimberg *et al*, 1998; Osterstrom *et al*, 2002; Murakami *et al*, 2005). These data obtained in humans differ from those obtained in mice, which showed an increased expression of *PLA2G2A* and no variation in *PLA2G5* expression in tumours from azoxymethane-treated mice (Ilsley *et al*, 2005).

### Expression level of genes involved in inflammation and tumorigenesis in colon adenocarcinomas and normal human colon

No significant changes between adenocarcinomas and normal colon were observed for most inflammation-related genes. It should be noted that many of these genes were expressed at high levels within the normal mucosa, in particular, *MMP-9*, *PLA2G6* and *ptgs2*. A recent study by Chen *et al* (2004) has also shown high mRNA expression levels of several inflammation-related genes in normal-appearing colon mucosa of patients with colon cancer. Therefore, our data would suggest a possible pre-existing inflammatory condition within the normal mucosa distant from the tumours, and likely explain the absence of increased expression in adenocarcinomas *vs* normal mucosa for several inflammation-related genes. The slight decrease in *ptgs1* (the COX-1 gene) appeared in agreement with that observed in colon adenocarcinomas of stage III (Duke's C) patients (Church *et al*, 2004). The COX-1 protein is considered to exert dual opposing effects in cancer, acting as either a tumour suppressor or a tumour initiator (Chulada *et al*, 2000). The slight decrease in *PLA2G4A* expression that we observed may be related to the dual opposing effects of cPLA_2_-IVA in cancer, that is, proliferative effects via metabolism of AA into eicosanoids, such as PGE_2_, and antiproliferative effects via AA-dependent ceramide production leading to apoptosis (Ilsley *et al*, 2005). The absence of increase in *ptgs2* (the COX-2 gene) levels in our set of patients is in accordance with two recent qPCR studies also performed on patients with colon cancer (Church *et al*, 2004; Gustafsson *et al*, 2007), and may be related to the inflammatory status of the normal mucosa discussed above. The fact that we did not observe an increase of COX-2 at the mRNA level, while we detected an overexpression at the protein level by immunohistochemistry as previously reported (Wendum *et al*, 2003), is in line with the post-transcriptional regulation of COX-2 expression (Dixon *et al*, 2003). Therefore, mRNA and protein levels of COX-2 may not be closely linked, as recently proposed in colon cancer tissues (Gustafsson *et al*, 2007) and colon cancer cells (Dixon *et al*, 2003).

### Downregulation of sPLA_2_-IID and sPLA_2_-V expression in human colon adenocarcinomas

A marked decrease in the expression level of *PLA2G2D* and *PLA2G5* was observed in both left- and right-sided adenocarcinomas (Figure 2, 3 and 4). These variations were also observed at the protein level by immunohistochemical analyses. Indeed, sPLA_2_-IID and sPLA_2_-V were found to be present in epithelial cells of normal mucosa, and their expression was markedly reduced in tumours (Figure 6). The decreased expression of sPLA_2_-IID and sPLA_2_-V in tumours may suggest a protective role of these sPLA_2_s, as it has been proposed for sPLA_2_-IIA in mice (MacPhee *et al*, 1995; Cormier *et al*, 1997). A similar decrease in expression of sPLA_2_-IID and sPLA_2_-V was also described in gastric tissues with signet-ring cell carcinoma (Masuda *et al*, 2005a). Interestingly, the *PLA2G2A*, *PLA2G2C*, *PLA2G2D*, *PLA2G2E*, *PLA2G2F* and *PLA2G5* genes reside within the same region of human chromosome 1 at p35–36.1 (Valentin *et al*, 2000), a region frequently altered in colorectal cancer (Spirio *et al*, 1996). The decreased expression for *PLA2G2D* and *PLA2G5* appears to be linked (cluster II, Figure 5) to that observed for *PLA2G4A*, *ptgs1*, *SFRP-1* and *RLPA-1*, which have been proposed as tumour suppressor genes in colon cancer (Chulada *et al*, 2000; Shida *et al*, 2004; Suzuki *et al*, 2004; Dong *et al*, 2005).

### Upregulation of PLA2G3 and Wnt target genes in human colon adenocarcinomas

A marked increase in sPLA_2_-III expression level was observed in both left- and right-sided adenocarcinomas, a finding that was confirmed by immunohistochemical analysis. No or very weak sPLA_2_-III expression was observed in normal epithelial cells, but a robust expression was observed in tumours (Figure 6). Our findings are consistent with the recent observation that sPLA_2_-III can trigger the proliferation of human colon cancer cells *in vitro* (Murakami *et al*, 2005). Parallel to the increase in *PLA2G3* gene expression level, we observed an increase in the expression level of four target genes of the Wnt/*β*-cat/Tcf-4 pathway: *Il-8* (Levy *et al*, 2002), *HD-6* (also called defensin *α*6) (Kolligs *et al*, 2002), *MMP-7* (also called matrilysin) (Nelson *et al*, 2000) and *EPR-1* (also called survivin) (Kolligs *et al*, 2002), as well as a concomitant dramatic decrease in *SFRP-1* gene expression level. Therefore, our data are consistent with the activation of the Wnt/*β*-cat/Tcf-4 pathway in the development of colon adenocarcinomas. It will be of interest to determine whether the expression of *PLA2G3* is related to the activation of the Wnt/*β*-cat/Tcf-4 pathway, as already suggested for *PLA2G2A* in gastric cancer (Aggarwal *et al*, 2006).

Our data further support the role of LPA in the pathogenesis of colon cancer. LPA exerts its effects through at least three different receptors: RLPA-1, RLPA-2 and RLPA-3 (Mills and Moolenaar, 2003). It has been demonstrated that the Wnt/*β*-cat/Tcf-4 pathway is involved in the proliferative effects of LPA through binding to RLPA-2 (Yang *et al*, 2005). We observed a decreased expression of *RLPA-1* and a sustained expression of *RLPA-2*, which is in agreement with the predominant expression of *RLPA-2* in adenocarcinomas (Shida *et al*, 2004). Whether sPLA_2_-III plays a role in the production of LPA, which in turn activates RLPA-2, remains to be established.

### The sPLA_2_-III as a novel potential biomarker of human colon cancer

Because of their upregulation during colorectal carcinogenesis, Wnt target genes and their associated products have been examined for their potential use as biomarkers. The level of Il-8 protein was found to be increased in colorectal cancer patients (Terada *et al*, 2005). A significant increase in the level of defensin *α*6 has also been found in patients with colon cancer (Nam *et al*, 2005). Although Il-8 and defensin *α*6 levels have been recently proposed as markers of human colorectal cancer (Nam *et al*, 2005; Terada *et al*, 2005), our data show that they would detect only left-sided adenocarcinomas (Figure 3, 4 and 5). In contrast, the increased expression of sPLA_2_-III was observed in both left and right colon adenocarcinomas, indicating that the analysis of sPLA_2_-III levels would detect cancers located in both left and right colon. MMP-7 is a matrix metalloprotease that has been associated with tumour invasion and metastasis (Nelson *et al*, 2000). We found that the expression pattern of *PLA2G3* was more similar to that of *MMP-7*, which was also increased in both left and right colon adenocarcinomas (Figure 3, 4 and 5).

In conclusion, this work is the first comprehensive analysis of the expression pattern of the full set of sPLA_2_s in human colon cancer. The distinct expression pattern observed for sPLA_2_ genes suggests that mRNA profiling of the full set of human sPLA_2_s may be useful to detect colon tumours either by analysing their expression pattern in tumours (Barrier *et al*, 2005), in circulating blood cells (Burczynski *et al*, 2005) or directly in serum on circulating mRNA, as recently proposed for other genes (Li *et al*, 2006). Moreover, we have observed a dramatic increase in sPLA_2_-III expression in both left- and right-sided adenocarcinomas, suggesting that sPLA_2_-III may represent a novel broad molecular biomarker of colon cancer. It will be of interest to determine whether the expression level of sPLA_2_-III also increases at earlier stages of tumorigenesis, including adenomas. It will also be useful to detect the sPLA_2_-III protein in human colon biopsies, stools or serum using the recently time-resolved fluoroimmunoassays developed for the different human sPLA_2_s (Nevalainen *et al*, 2005).
